# Pregnancy-related acquired hemophilia A initially manifesting as pleural hemorrhage: A case report

**DOI:** 10.1097/MD.0000000000014119

**Published:** 2019-01-18

**Authors:** Lili Qian, Hangping Ge, Pingping Hu, Ni Zhu, Junfa Chen, Jianping Shen, Yu Zhang

**Affiliations:** a Department of Laboratory; a Department of Laboratory, b Department of Hematology, First Affiliated Hospital of Zhejiang Chinese Medical University, Hangzhou, China.; b Department of Hematology, First Affiliated Hospital of Zhejiang, Chinese Medical University, Hangzhou, China.

**Keywords:** acquired hemophilia A, hemothorax, pregnancy

## Abstract

**Rationale:**

Acquired hemophilia A is a rare hemorrhagic disease in which the body produces specific antibodies that attack factor VIII, resulting in bleeding that is mainly mucocutaneous and associated with soft tissue and the gastrointestinal system. Approximately 50% of this disease derives from basic diseases, such as autoimmune diseases, cancer, and pregnancy.

**Patient concerns:**

We report a 35-year-old postpartum female with acquired hemophilia A who initially presented with pleural hemorrhage.

**Diagnoses:**

In this patient activated prothrombin time (PT) and activated partial thromboplastin time (APTT) were found, and the factor VIII activity was 12.6%, furthermore Bethesda assay showed a FVIII antibody titer of 7.4 Bethesda units (BUs).

**Interventions:**

The treatment requires a 2-pronged approach: treatment of the bleeding and elimination of the inhibitor.

**Outcomes:**

After hemostatic agents were used and inhibitors were eradicated, the patient achieved complete remission without relapse.

**Lessons:**

It is essential to recognize the development of disease earlier in pregnant woman.

## 1 Introduction

The incidence of acquired hemophilia A (AHA) is approximately 1 to 3 per million per year.^[1,2]^ Bleeding in AHA is often severe, with reported mortalities of 9% to 27%.^[3,4]^ Autoimmune diseases or postpartum conditions are most often associated with AHA in young individuals. In the elderly, a link between cancer and/or concomitant drug use and AHA has been recognized. A 35-year-old postpartum woman presented with pleural hemorrhage and was finally diagnosed with AHA. The patient achieved complete remission after treatment with activated prothrombin complex concentrate (aPCC), human factor VIII (hFVIII) concentrates, corticosteroids, and plasma. She is currently undergoing a 6-month follow-up and has shown no recurrence.

## 2 Case

**Figure 1:**
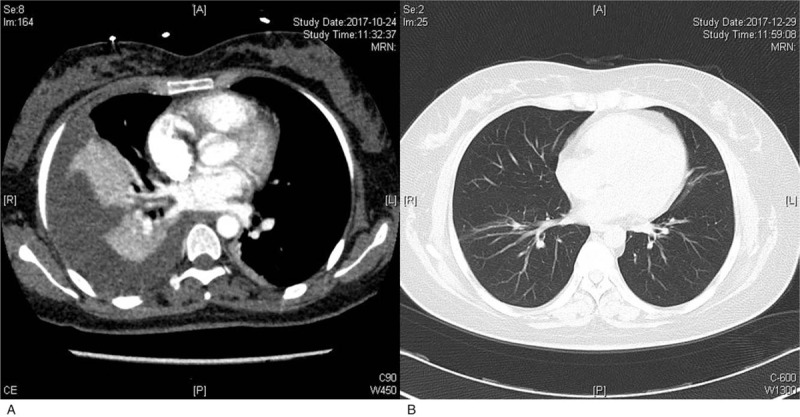
The changes of pulmonary computed tomography images. A: Computed tomography angiography of the aorta showed a large amount of pleural effusion in the right thoracic cavity and partial right pulmonary collapse; B: At the 2-month follow-up visit, her pulmonary computed tomography revealed that the pleural hemorrhage had subsided.

A 35-year-old woman who presented with a 5-day history of chest tightness and right leg pain was admitted to our emergency department on October 22, 2017. The patient had delivered (first pregnancy) 48 days prior and had an unremarkable medical history. Upon physical examination, dullness to percussion was noted over her right lower lung. Swelling, tenderness, and ecchymosis were present in the right medial thigh. The circumference of the right thigh was 53.5 cm, while that of the left thigh was 49 cm. Computed tomography angiography of the aorta showed a large amount of pleural effusion in the right thoracic cavity and partial right pulmonary collapse (see Fig. 1A). Under B-mode ultrasound guidance, thoracentesis was performed, and bloody pleural effusions were drained. Her white blood cell count was 17.9 (10^9^/L), with 75.8% neutrophils; hemoglobin was 70 (g/L), and platelets were 238 (10^9^/L). Prothrombin time (PT) was 15.20 s, and activated partial thromboplastin time (APTT) was 68.40 s. An APTT mixing study showed that her APTTs were 70.12 s, 30.45 s, and 60.40 s at 0, 1, and 2 h, respectively. Factor IX activity was 107.8 (%), factor XI activity was 66.9%, and factor VIII activity was 12.6%. The Bethesda assay showed a FVIII antibody titer of 7.4 Bethesda units (BUs). The diagnosis of AHA was confirmed.

The regimen for this patient included aPCC (10 U/kg intravenously 3 times daily), hFVIII (20 IU/kg intravenously twice daily), prednisone (1 mg/kg orally once daily), and plasma (400 mL intravenously once daily). Two weeks later, the ecchymosis in her medial thigh improved, and PT and APTT were 17.70 s and 20.30 s, respectively. FVIII activity was 127.30%, and the FVIII antibody titer was 0 BU. After prednisone was tapered to 10 mg orally once daily, the patient was discharged. At the 2-month follow-up visit, her pulmonary computed tomography revealed that the pleural hemorrhage had subsided (see Fig. 1B). Prednisone was withdrawn at a rate of 20% every 2 weeks. The patient is now undergoing 6-month follow-up and has shown no recurrence.

## 3 Discussion

Pregnancy-related AHA accounts for 7% to 11% of cases of this disease and is most common within 1 to 4 months after delivery.^[5,6]^ In very few cases, an inhibitor appears during pregnancy.^[7]^ The potency of the antibody is rather low in the majority of cases, and the overall prognosis of pregnancy-related AHA is good; however, future pregnancies may lead to a recurrence of AHA.^[8]^ AHA mainly manifests as hemorrhages in the skin, mucous membranes, muscles, joints and gastrointestinal tract. In our case, the patient had delivered (first pregnancy) 48 days prior, and with an initial presentation of pleural effusion as the main manifestation, which is rarely reported in other cases. Therefore in future clinical work, the diagnosis of secondary hemophilia should be taken into consideration in women with abnormal coagulation function accompanied by pleural effusion and pregnancy history.

The goals of AHA treatment are to control the bleeding and suppress the inhibitor. First-line hemostatic treatment includes bypassing agents: recombinant factor VIIa (rFVIIa) and aPCC.^[9,10]^ In case of low-titer inhibitors, hFVIII concentrates can also be used.^[11]^ The methods for removing antibodies include administration of corticosteroids, cyclophosphamide, rituximab, intravenous immunoglobulin, and plasmapheresis/immunoadsorption and the induction of immune tolerance.^[12,13]^ Treatment regimens should aim to balance the need to quickly eradicate the inhibitor and reduce exposure to the side effects of immunosuppressive therapy.^[14]^ Thus, we used prednisone alone, and no evidence is needed to confirm that cyclophosphamide and steroids are superior to steroids alone.^[15,16]^ Unfortunately, our patient did not receive treatment with rFVIIa but, rather, with aPCC and hFVIII concentrates due to costs. This case study will help to raise our awareness of the diagnosis and early treatment of AHA.

## Author contributions

**Conceptualization:** Lili Qian.

**Investigation:** Ni Zhu.

**Writing – original draft:** Hangping Ge, Pingping Hu, Junfa Chen.

**Writing – review & editing:** Jianping Shen, Yu Zhang.

Yu Zhang orcid: 0000-0002-4230-6919.

## References

[1] Borg JY Guillet B Le Cam-Duchez V Outcome of acquired haemophilia in France: the prospective SACHA (Surveillance des Auto antiCorps au cours de l’Hemophilie Acquise) registry [J]. Haemophilia 2013;19:564–70. 23574453 10.1111/hae.12138

[2] Collins P Macartney N Davies R A population based, unselected, consecutive cohort of patients with acquired haemophilia A. Br J Haematol 2004;124:86–90. 14675412 10.1046/j.1365-2141.2003.04731.x

[3] Knoebl P Baudo F Collins PW Management of bleeding in acquired hemophilia: results of the European Acquired Hemophilia Registry (EACH2). Blood 2010;116:315–6.

[4] Knoebl P Marco P Baudo F Demographic and clinical data in acquired hemophilia A: results from the European Acquired Haemophilia Registry (EACH2). J Thromb Haemost 2012;10:622–31. 22321904 10.1111/j.1538-7836.2012.04654.x

[5] Bossi P Cabane J Ninet J Acquired hemophilia due to factor VIII inhibitors in 34 patients. Am J Med 1998;105:400–8. 9831424 10.1016/s0002-9343(98)00289-7

[6] Michiels JJ Acquired hemophilia A in women postpartum: clinical manifestations, diagnosis, and treatment. Clin Appl Thromb Hemost 2000;6:82–6. 10775027 10.1177/107602960000600206

[7] Baudo F De Cataldo F Italian association of haemophilia centres: register of acquired factor V I. Acquired factor VIII inhibitors in pregnancy: data from the Italian Haemophilia Register relevant to clinical practice. BJOG 2003;110:311–4. 12628274 10.1016/s1470-0328(03)01935-9

[8] Solymoss S Postpartum acquired factor VIII inhibitors: results of a survey. Am J Hematol 1998;59:1–4. 9723568 10.1002/(sici)1096-8652(199809)59:1<1::aid-ajh1>3.0.co;2-t

[9] Delgado J Jimenez-Yuste V Hernandez-Navarro F Acquired haemophilia: review and meta-analysis focused on therapy and prognostic factors. Br J Haematol 2003;121:21–35. 12670328 10.1046/j.1365-2141.2003.04162.x

[10] Lak M Sharifian RA Karimi K Acquired hemophilia A: clinical features, surgery and treatment of 34 cases, and experience of using recombinant factor VIIa. Clin Appl Thromb Hemost 2010;16:294–300. 19211581 10.1177/1076029608331227

[11] Cugno M Gualtierotti R Tedeschi A Autoantibodies to coagulation factors: from pathophysiology to diagnosis and therapy. Autoimmun Rev 2014;13:40–8. 23954454 10.1016/j.autrev.2013.08.001

[12] Shetty S Bhave M Ghosh K Acquired hemophilia a: diagnosis, aetiology, clinical spectrum and treatment options. Autoimmun Rev 2011;10:311–6. 21115138 10.1016/j.autrev.2010.11.005

[13] Ugur Bilgin A Ozcan M Ayyildiz E The treatment of acquired hemophilia with combination therapy of immunosuppressives and immunoadsorption. Turk J Haematol 2014;31:194–6. 25035681 10.4274/tjh.2013.0178 PMC4102051

[14] Baudo F László Nemes Pellegrini F Immunosuppression for acquired hemophilia A: results from the European Acquired Haemophilia Registry (EACH2). Blood 2012;120:47–55. 22517903 10.1182/blood-2012-02-409185 PMC3390961

[15] Green D Rademaker AW Briet E A prospective, randomized trial of prednisone and cyclophosphamide in the treatment of patients with factor VIII autoantibodies. Thromb Haemost 1993;70:753–7. 8128430

[16] Collins PW Hirsch S Baglin TP Acquired hemophilia A in the United Kingdom: a 2-year national surveillance study by the United Kingdom Haemophilia Centre Doctors’ Organisation. Blood 2007;109:1870–7. 17047148 10.1182/blood-2006-06-029850

